# Mechanism and application of fibrous proteins in diabetic wound healing: a literature review

**DOI:** 10.3389/fendo.2024.1430543

**Published:** 2024-07-26

**Authors:** Lilin Yan, Yuqing Wang, Jiawei Feng, Yiming Ni, Ting Zhang, Yemin Cao, Mingmei Zhou, Cheng Zhao

**Affiliations:** ^1^ Shanghai Traditional Chinese Medicine Integrated Hospital, Shanghai University of Traditional Chinese Medicine, Shanghai, China; ^2^ Institute of Interdisciplinary Integrative Medicine Research, Shanghai University of Traditional Chinese Medicine, Shanghai, China

**Keywords:** diabetic foot ulcers, fibrous proteins, extracellular matrix, wound healing, wound dressing

## Abstract

Diabetic wounds are more complex than normal chronic wounds because of factors such as hypoxia, reduced local angiogenesis, and prolonged inflammation phase. Fibrous proteins, including collagen, fibrin, laminin, fibronectin, elastin etc., possess excellent inherent properties that make them highly advantageous in the area of wound healing. Accumulating evidence suggests that they contribute to the healing process of diabetic wounds by facilitating the repair and remodel of extracellular matrix, stimulating the development of vascular and granulation tissue, and so on. However, there is currently a lack of a comprehensive review of the application of these proteins in diabetes wounds. An overview of fibrous protein characteristics and the alterations linked to diabetic wounds is given in this article’s initial section. Next is a summary of the advanced applications of fibrous proteins in the last five years, including acellular dermal matrix, hydrogel, foam, scaffold, and electrospun nanofibrous membrane. These dressings have the ability to actively promote healing in addition to just covering wounds compared to traditional wound dressings like gauze or bandage. Research on fibrous proteins and their role in diabetic wound healing may result in novel therapeutic modalities that lower the incidence of diabetic wounds and thereby enhance the health of diabetic patients.

## 1 Introduction

Diabetic foot ulcers (DFUs) are a prevalent and severe result of prolonged diabetes mismanagement, impacting around 18.6 million individuals globally annually. Approximately 20% of individuals with DFU need lower limb amputation, either mild (below the ankle), severe (above the ankle), or both, and 10% die within a year of being diagnosed with the DFU (1). In addition, patients with DFU have worse health-related quality of life, less psychosocial adaptability, and a higher burden of health care encounters (2). The direct costs of treating diabetic foot ulcers are estimated to range from $9 billion to $13 billion yearly (3). DFU can be attributed to multiple reasons such as nerve injury, reduced blood circulation, and changes in the release of growth factors (4). Despite advancements in technology like biogenesis of skin cells and the common use of standard care for diabetic wound treatment, wound healing rates have been reported to be less than 50% (5). New methods are needed to enhance the effectiveness of treatments.

Up to now, various natural materials have been used to improve the diabetic wound healing process. Fibrous proteins such as collagen, elastin, fibronectin, fibrin and laminin offer a range of therapeutical benefits for wound healing. They have the ability to bind and control the availability of growth factors, acting as a storage system for growth factors, provide structure support and promote cell survival, differentiation, adhesion and proliferation (6). It has been proved that fibrous proteins can promote the healing of diabetic wounds by regulating the structural and mechanical properties of extracellular matrix (ECM) and influencing cellular activity as extracellular signaling molecules (7). In recent years, the above proteins are more used combined with wound dressings to better promote wound healing.

Dressings are essential for wound management and care. Wound dressings primarily serve to offer a temporary physical barrier that absorbs wound drainage and maintains the required moisture environment to enhance wound re-epithelialization. Due to defects in their material properties, traditional wound dressings (e.g., gauze) unfortunately have limited basic functionality (8). Additionally, microangiopathy in diabetic patients can reduce oxygen and blood supply to the wound bed, which can delay wound healing and increase the risk of infection (9). Therefore, there is a need for dressings with good hemostatic maintenance capability, anti-infection and pro-repair ability to treat diabetic wounds (10). Modern wound dressings have become the primary choice for the treatment of various types of wounds due to their biocompatibility and biodegradability. In addition, they are able to maintain sufficient temperature and humidity in the treatment environment to relieve pain, improve the hypoxic environment, and stimulate wound healing (11). Therefore, fibrous proteins are more used in combination with modern wound dressings, which can not only provide a moist environment for wounds, but also activates some endogenous signaling pathways, accelerates cell proliferation and migration, and achieves the purpose of promoting wound healing.

This article aims to provide an overview of the potential of fibrous proteins in diabetic wound healing and related mechanisms and focus on fibrous proteins-based modern wound dressings that promote the complex process of diabetic wound healing and its associated mechanisms. First, the differences between normal wounds and diabetic wounds in the healing process were summarized; secondly, recent advances in the study of fibrous proteins were then reviewed; thirdly, application of fibrous proteins in combination with modern wound dressings was introduced. We believe that this review is able to offer scientific knowledge and recommendations for the further treatment of diabetic wounds and thus reduce the occurrence of amputation in people with diabetes.

## 2 The healing process of wounds

### 2.1 Normal wound healing

**Figure 1:**
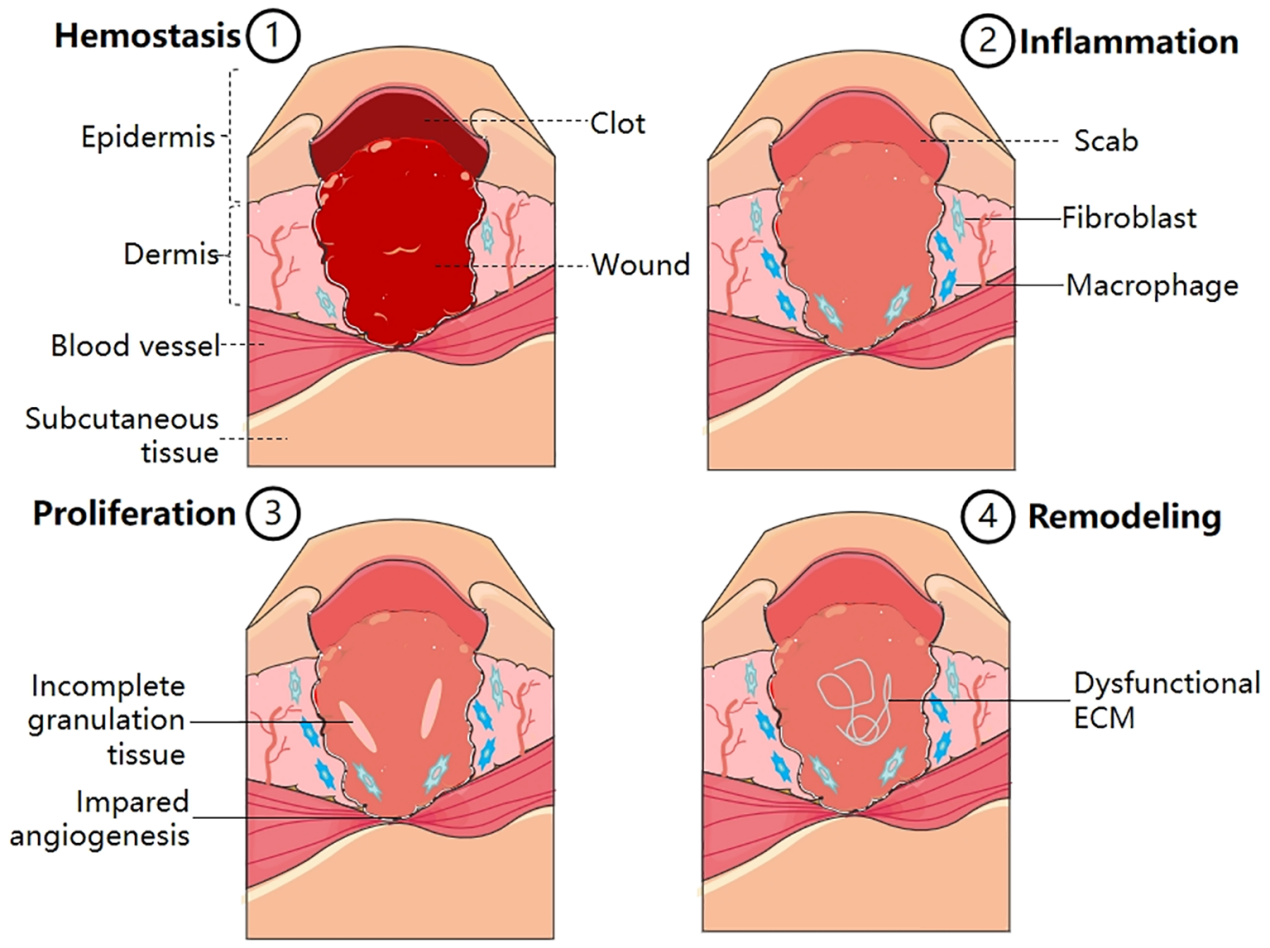
Normal wound healing process, consisting of four main stages–hemostasis, inflammation, proliferation and remodeling (1). Hemostasis: Platelet activation; Fibrin clot formation; Thrombosis (2). Inflammation: Release of inflammatory mediators; Transformation of monocytes into macrophages (3). Proliferation: Formation of granulation tissue; Formation of fibrous tissue; Epithelial cell proliferation (4). Remodeling: Collagen reorganization; ECM reconstruction.

Wound healing is a complex and continuous process that involves four procedurally precise phases: hemostasis, inflammation, proliferation, and remodeling (12) (**Figure 1**). Events at each stage must be executed accurately and regularly. Interruption, abnormality, or prolongation of any process can lead to delayed wound healing or chronic wounds that do not heal (13). The hemostasis phase, which happens immediately after an injury, involves the formation of a fibrin and platelet plug, which triggers a coagulation cascade, and then helps to stop bleeding at the injury site. Additionally, it aids in the recruitment of cells from the surrounding tissue and circulation (14). During the inflammatory phase, which typically lasts for 1-3 days, mast cells release inflammatory mediators like 5-hydroxytryptamine (5-HT) and histamine, which increase the blood vessel permeability at the wound site and promote the migration of neutrophils, monocytes, and chemokines to the site of injury, leading to an inflammatory response (15). Among them, monocytes that go to the wound tissue undergo differentiation into macrophages in response to the specific local environment (16). Furthermore, inflammatory mediators and cells are essential for removal of necrotic tissue and foreign bodies, as well as initiating and controlling the wound healing process (17). During the proliferative phase (4-21 days) of wound healing, the M2 macrophage population takes on a corresponding phenotype, which is marked by the release of various growth factors (GFs) including platelet-derived growth factors (PDGFs), tumor growth factor β1 (TGF-β1), vascular endothelial growth factor α (VEGF-α), platelet factor 4 (PF4) etc. (18). These chemotactic factors stimulate fibroblasts, endothelial cells and keratinocytes in surrounding tissues to initiate migration and proliferation. Among these cells, fibroblasts play a crucial role in producing and depositing new ECM to restore the skin’s structural integrity. When exposed to mechanical tension and GFs, subpopulations of fibroblasts brought to a wound site transform into myofibroblasts (19). These myofibroblasts are key players in the healing process, contributing to creating scar tissue through the synthesis and arrangement of collagen and ECM components (20), which eventually creates a protective barrier between the wound and the surrounding environment, ultimately leading to wound closure (21). Remodeling, which occurs after 21 days or more, is the final stage of wound healing. Significantly, the ECM undergoes dynamic alterations during this period, leading to the maturation of its structure (22).

### 2.2 Diabetic wound healing

**Figure 2:**
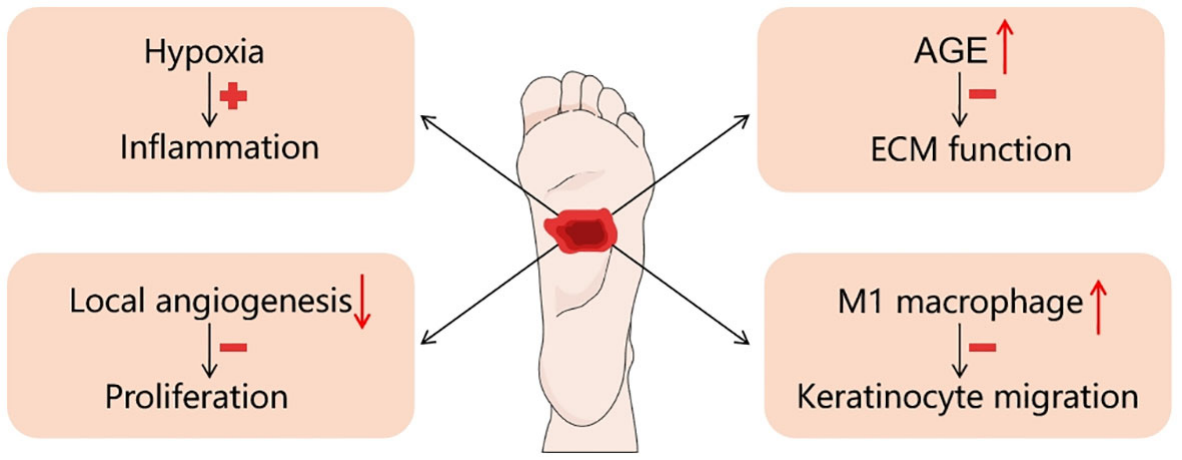
Factors affecting diabetic wound healing.

Diabetic wounds are formed when persistent stimulation or abnormal factors hinder wound recovery. They develop into chronic wounds that exhibit difficulty to healing due to many factors (**Figure 2**).

Firstly, hypoxia is a primary factor contributing to the difficulty in diabetic wound healing. Oxygen is an important regulator of wound healing processes such as skin cell proliferation, granulation, re-epithelialization, angiogenesis, and tissue regeneration (23). However, after an injury, the damaged area experiences a rise in hypoxia due to disrupted blood flow and higher oxygen consumption caused by inflammatory reactions, along with a decrease in oxygen supply (24). This leads to a significant decrease in oxygen usage, resulting in hypoxia related to high glucose levels (25). Clinical studies have shown that the hyperoxidative environment associated with hyperglycemia and tissue hypoxia can infiltrate unhealed diabetic wounds, leading to delayed wound repair (26). Hypoxia also prolongs the effective healing time of wounds by increasing the inflammatory response and increasing oxygen radical levels. In one literature, a sustained oxygenation system was prepared, comprising of microspheres that release oxygen and a hydrogel that scavenges reactive oxygen species (ROS). It was proved to accelerate healing process by continuous oxygenation and ROS scavenging. Specifically, it enhanced the proliferation phase, and decrease of the proinflammatory cytokine expression (27).

Secondly, diabetic wound is characterized by a reduction in the generation of local angiogenesis. It depends on maintaining a delicate balance between stimulating the growth and proliferation of blood vessels and promoting their maturity and quiescence in the process of normal wound healing, while the pathophysiology of diabetes severely disrupts this balance, inhibiting normal wound healing and tissue regeneration. Studies showed that VEGF-A protein (28) and PDGF (29) were significantly reduced in wounds in db/db mice compared to normal controls (30), suggesting that diabetes reduces angiogenesis during wound healing, making proliferation more difficult. Therefore, promoting diabetic wound healing by enhancing angiogenesis at the wound site has become a popular avenue.

Thirdly, unlike normal wound healing, diabetic wounds are characterized by the non-resolving inflammation phase, where a large number of macrophages are found (31). In diabetic wounds, polarization of M1 (pro-inflammatory) macrophages is abnormally regulated and persistent, whereas in normal wounds, macrophages transform into M2 (pro-healing) macrophages around day 3 post-injury (32). In addition to M1 macrophages, myelopoiesis in hematopoietic stem cells has been reported to increase due to diabetic microenvironment, leading to elevated levels of circulating blood Ly6C^Hi^ monocytes, which invade wounds and differentiate into macrophages with M1-like properties, including Interleukin-1β (IL-1β) and tumor necrosis factor-α (TNF-α) (33), both of which inhibit keratinocyte migration and slow wound healing.

Fourthly, abnormalities in ECM accumulation and remodeling primarily affect the proliferative and remodeling phases of diabetic wound healing. The ECM primarily serves as a supporting factor in the process of wound healing. However, the structures and functions of ECM in diabetic wounds are significantly compromised as a result of fibroblast dysfunction and the heightened gaps between collagen fibers (34). What is more, high glucose level makes ECM proliferation, thickening and glycosylation (35). It also leads to the production of advanced glycation end-products (AGEs), which are frequently detected in the ECM proteins of individuals with diabetes. Consequently, the mechanical support provided by ECM fibrils to capillary buds during vasculogenesis in diabetic wounds is affected (36). These effects can lead to abnormal structure and function of ECM, resulting in slow wound healing.

In conclusion, diabetic wounds face a greater challenge for healing compared to other wounds. They differ from other wounds in that they exhibit hypoxia, impaired angiogenesis, chronic inflammation and abnormal ECM, resulting in a slower healing process. We found that fibrous proteins promote diabetic wound healing, so related proteins and their dressings can be used to address this issue.

## 3 Effects of fibrous proteins on diabetic wound healing

**Figure 3:**
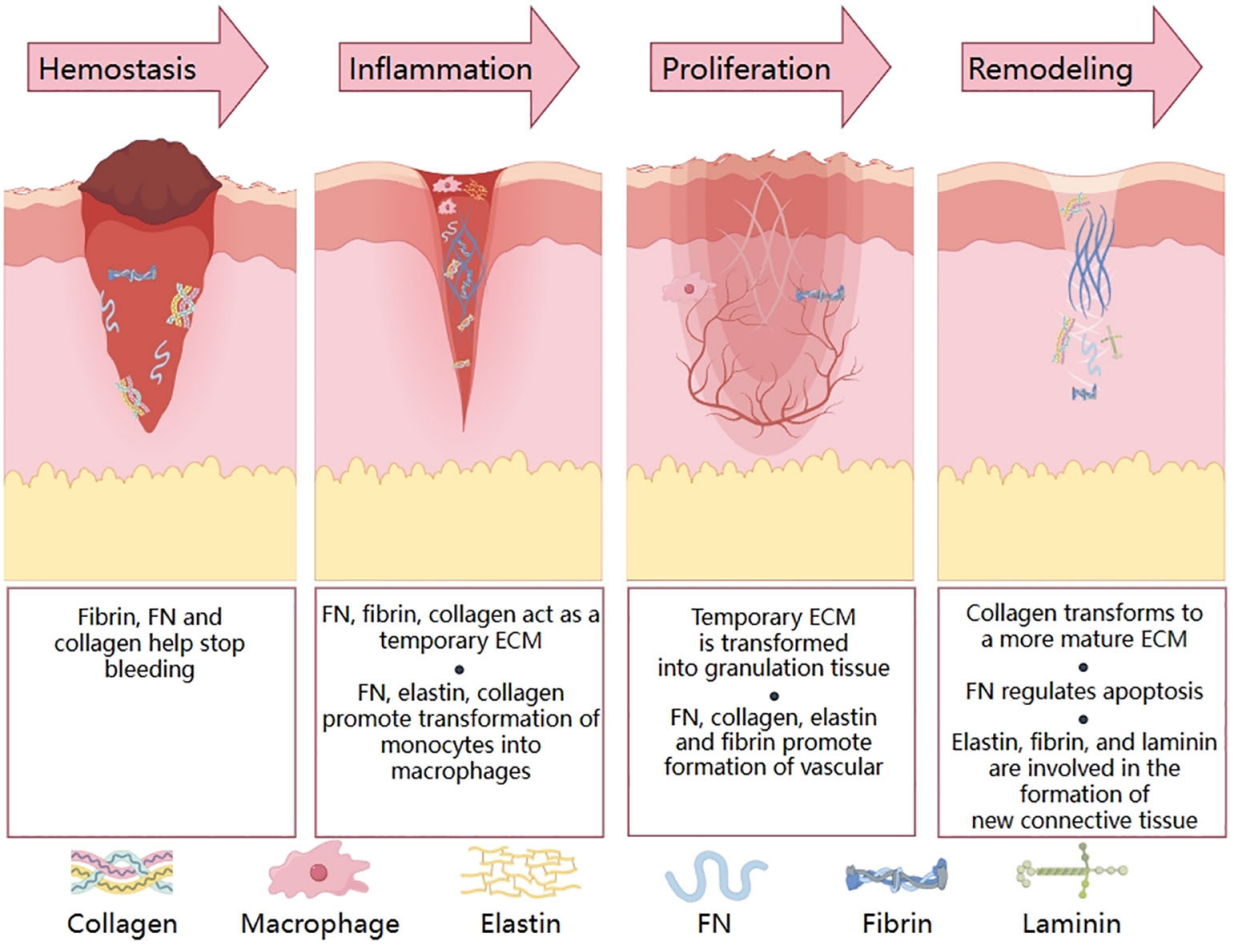
Functions of fibrous proteins in four stages of normal wound healing.

Fibrous proteins include collagen, elastin, fibronectin, laminin and fibrin. Collagen is the main component of ECM. Elastin helps to determine the normal rigidity and elasticity of the skin, and the latter three proteins are important mediators of hemostasis and cell migration during wound healing (**Figure 3**).

### 3.1 Collagen

**Figure 4:**
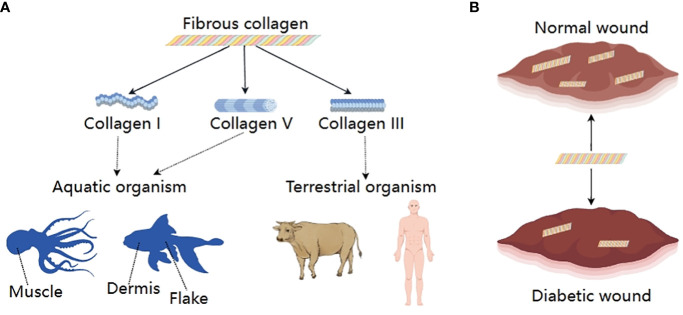
Fibrous collagens; **(A)** Classification and sources, **(B)** Changes between normal wound and diabetic wound.

Collagens are the main protein component of the ECM. The collagen family consists of 28 members, which contain at least one triple-helical domain. Some collagens have specific biological functions due to their restricted tissue distribution (37). Based on their respective roles, they can be categorized into fibrous collagen and non-fibrous collagen. Among them. the fibrous collagens consist of collagen types I, III, and V. They are extensively and mostly present in tissues and contribute to the tensile strength (7). Type I collagen is the major protein of bone, skin, and tendons, while type III collagen, along with type I collagen, is the major structural component of blood vessels (38) (**Figure 4**).

The expression, deposition, and remodeling of collagen are critical in wound healing. In the phase of inflammation, collagen crosslinked fibrin together with aggregated platelets help stop bleeding. In addition, the crucial function of collagen in promoting M2 macrophage phenotype that is anti-inflammatory and pro-angiogenic through micro RNA signaling pathways has been proven (38). During the early proliferative phase, collagen type III binds with other ECM proteins such as fibronectin and tenascin to form a transient structure that provides support for cellular activities. Subsequently, the ECM consists primarily of collagen I and experiences further development as it goes through the remodeling process (39). Nevertheless, the healing process of diabetic wounds is impeded by aberrant collagen metabolism. According to a clinical investigation, there was a decrease in collagen deposition observed in individuals diagnosed with type I diabetes mellitus. Animal models also showed hyperglycemic environment can significantly increase levels of collagenase activity in rodent (40). In rats with diabetes induced by streptozotocin (STZ), catabolic processes of collagen formed during the diabetic state or before both were enhanced in rat skins.

### 3.2 Fibronectin

Fibronectin (FN) is an essential ECM glycoprotein involved in every stage of wound healing. Based on its distribution, it has been traditionally separated into two well-researched categories: plasma soluble FN (pFN) and cellular derived FN (cFN). The two demonstrate differences in function. pFN plays a key role in the early stages of wound healing by aiding in clot formation and facilitating the formation of a cell-extracellular matrix structure following injury. It also promotes epithelialization and the formation of granulation tissue. However, in diabetic wound tissues, pFN is degraded as a result of increased proteolytic activity. Thus, levels of pFN were found decrease in diabetic patients. cFN is then synthesized by the cells, including macrophages, fibroblasts, and possibly endothelial cells (41), migrates into the clot, and controls the later phases of tissue restructuring through the assembly of FN expressed locally (42, 43). In diabetic patients, plasma levels of cFN were elevated, which may result from changes in polarized secretion in diabetes (44). Furthermore, a previous study demonstrated that FN is significantly increased in the initial wound matrix and is high in diabetic tissues (45). Experiments to promote wound healing have been shown to be associated with an increase in FN.

**Figure 5:**
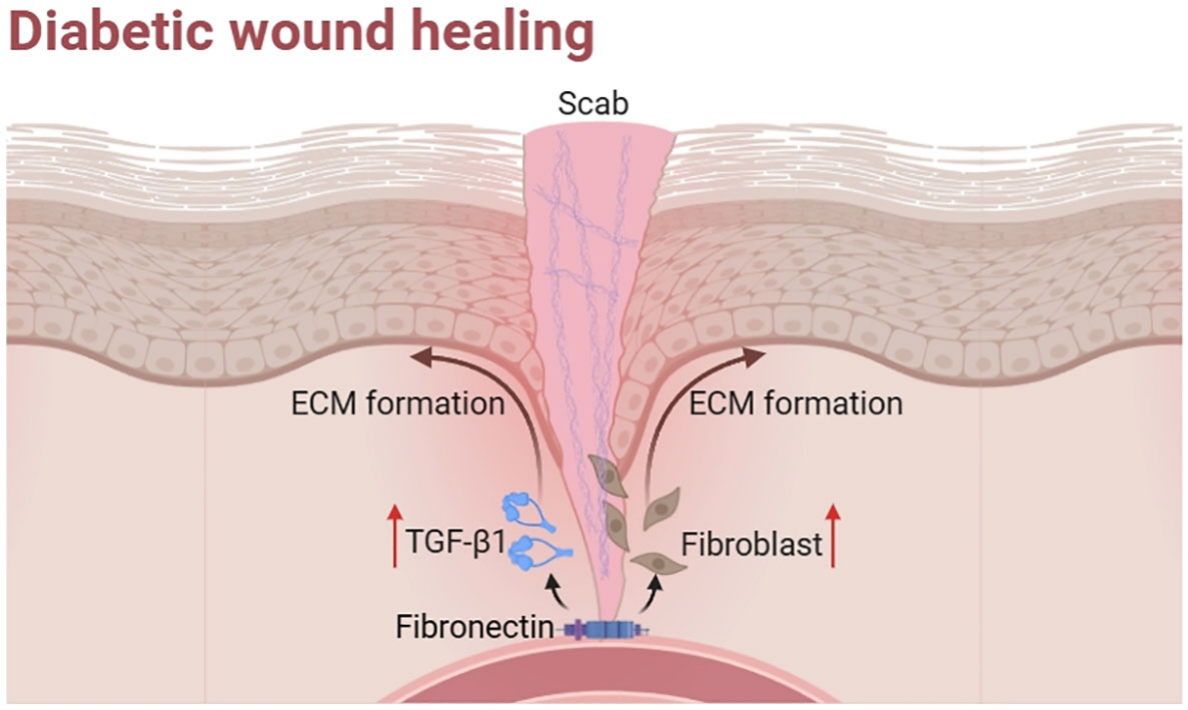
Functions of fibronection in diabetic wound healing.

Studies have shown that FN and its mimetics can promote wound healing in diabetes (**Figure 5**). For example, pFN applied as a topical therapy may enhance diabetic wound healing in rats by boosting fibroblast activity and promoting the production of TGF-β1 (46). Similarly, fibronectin matrix mimetics were shown to accelerate wound closure and promote the uniform distribution of collagen-rich granulation tissue (47). Therefore, the application of fibronectin matrix mimetics can serve as a supplementary component to different therapeutic strategies, thereby augmenting the wound healing process for chronic conditions. It is noteworthy that in the process of wound healing, soluble fibronectin is transformed into active, insoluble fibrils via a cell-mediated mechanism. Aiming to overcome decreased fibronectin fibril formation and its impact on wound healing capacity, a chimeric fibronectin fragment was synthesized and evaluated to imitate the insoluble, fibrous structure of a naturally existing ECM protein that plays a role in the process of wound healing. They found this fragment accelerated wound closure and promoted granulation tissue formation in diabetic mice (48). Collectively, research on animal models indicates that the disorders of fibronectin production and degradation contribute to impaired healing.

### 3.3 Elastin

Elastin is a crucial protein constituent of ECM in many tissues that require elasticity for their proper functioning, such as blood vessels, lung, and skin (49). It is classically considered the primary elastic component of elastic fibers and that undergoes extensive crosslinking to establish a stable structure (50), thus it plays a vital role in the structure and function of skin. It has been reported that elastin could not only modulate cellular behavior in various types of cells to produce biological reaction, which include fibroblast migration, proliferation, keratinocyte migration and promoting an angiogenic phenotype in endothelial cells but also alter cell activity, which has implications in wound healing (51). Compared to normal intact skin, elastin is significantly reduced in the upper dermis throughout the dermis of venous leg ulcer biopsies and DFU, but in DFU, the loss was throughout the dermis (52). Furthermore, patients with T2D exhibited increased inflammatory-mediated degradation of vascular elastin (53).

### 3.4 Laminin

Laminins (LMs), one of the major components of basement membrane (BM), are a large family of high molecular weight glycoproteins (54). They have been shown to have a positive impact on wound healing. For example, LM411 is the basic LM found in the BMs of capillaries and larger blood vessels. It has been proven to play a role in controlling endothelial cell survival, movement, and attachment. Most studies focus on the laminin isoforms rather than the overall expression level of laminin in diabetes. It is reported that LMs were increased in rats with diabetes compared with normal subjects (55). Furthermore, immunofluorescence micrographs shows that compared to normal rats, the fluorescence intensity was significantly higher in the unwounded corneas of diabetic rats. The delayed expression of LMs and its fragmented and irregular deposition were seen in the injured corneas. The findings of this study suggest a potential association between delayed wound healing in individuals with diabetes and the delayed reappearance and abnormal reformation of LMs (56).

### 3.5 Fibrin

**Figure 6:**
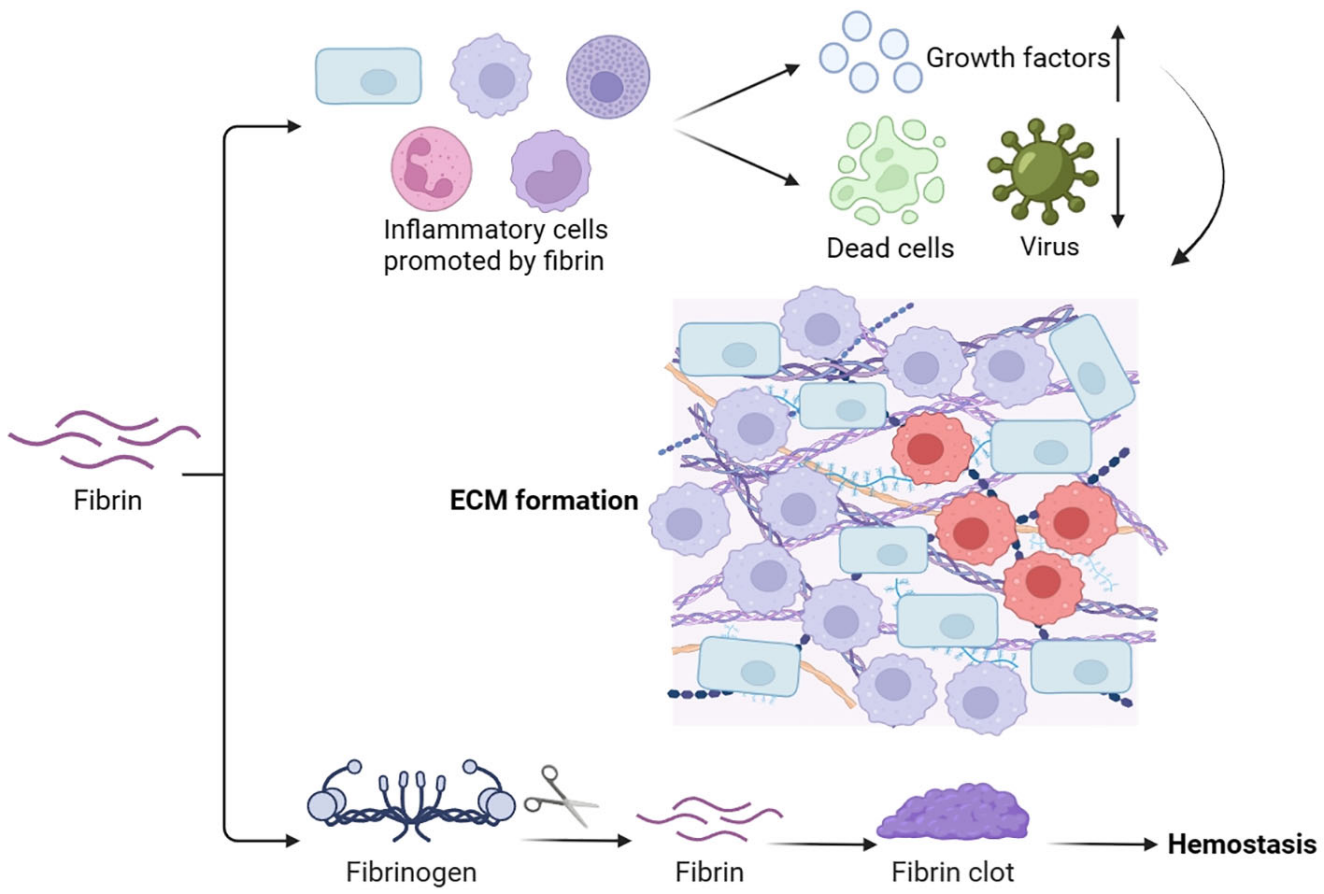
Mechanisms of fibrin in wound healing.

Fibrin is derived from the soluble plasma protein fibrinogen, which is produced in the liver and present in the bloodstream. It plays a vital role in hemostasis, wound healing, inflammation, angiogenesis, and various other biological processes (57) (**Figure 6**). The main purpose of the fibrin matrix is to halt bleeding. During hemostasis, fibrinogen is converted to fibrin, forming fibrin clots, which act as hemostasis, block microbial invasion, and provide a matrix scaffold for cell attachment (58). Aside from its function in hemostasis, the fibrin matrix also promotes the attraction, migration, adhesion, and growth of several cells crucial for wound healing, including inflammatory cells (59). It has been observed that hyperglucagonemia in type 2 diabetes could lead to the elevated synthesis of plasma fibrinogen (39). Besides, type 2 diabetes patients may have modified kinetics in the formation of fibrin networks, leading to reduced pore size and lysis rate of fibrin clots. This suggests a distinction in the fibrin matrix between diabetic and normal wound healing (60).

## 4 Application of fibrous proteins in diabetic wound healing

Fibrous proteins are extensively employed as wound dressings in the management of diabetes due to their exceptional functions. The wound dressings that have been studied thus far are presented in **Table 1**. The primary focus lies on the materials utilized, as well as the mechanisms and distinctive characteristics involved in the process of diabetic wound healing.

**Table 1:** Application of fibrous proteins in diabetic wound healing.

Type of dressing	Fibrous proteins/source	Other materials	Properties	Mechanism of wound healing	Reference
**Hydrogel**	Bovine collagen	Fibroblasts	• Collagen hydrogel did not exhibit significant cytotoxic effects.	• It promotes collagen synthesis, angiogenesis, activates macrophages, stimulates epithelial development and increases the thickness, density, and elasticity of the skin in the wound area.	(61)
	Hyaluronan/collagen	High-sulfated hyaluronan	• sHA is released from the hydrogel without material degradation.	• Hydrogels reduce inflammatory function in tissue-resident and bone marrow derived macrophages and impair inflammatory crosstalk of macrophages.	(62)
	Chitosan/collagen	Silver Nanoparticles	• Fibrous, streaky structure and large pores.	• Gels application increase hair follicle repair, sebaceous glands formation and VEGF, TGF-β1, IL-1β and TIMP1 gene expression.	(63)
	Fibrinogen	Engineered VEGF/PDGF-BB proteins	• Fibrin gels are prepared with the maximum growth factor concentration that would not disrupt fibrin polymerization.	• Application of modified forms of VEGF and PDGF-BB on fibrin hydrogels promotes arteriogenesis, triggers robust angiogenesis, and controls vascular expansion.	(64)
	Fibrin/collagen	Stromal vascular fraction	• It has good biological compatibility and cell proliferation, migration, and vitality, suitable for skin organotypic cell culture.	• Skin thickness and density in the vascular beds of the hypodermis are increased.	(65)
	Laminin	Gelatin/sericin/ adipose-derived stem cell	• It owns antioxidant potential and free radical scavenging.	• The addition of sericin leads to the improved protective effect to cells under oxidative stress. • The hydrogel could promote attachment of endothelial cells onto and thus promote angiogenesis. • The deposition of collagen I is enhanced and reticular arrangement of collagen is observed.	(66)
**Foam**	Bovine collagen	Gentamicin sulfate	• Biocompatible	• Antibacterial activity against many strains of gram-negative and gram-positive pathogens.	(67, 68)
	Collagen	Skin-derived precursors	• Greater cell viability	• SKPs and collagen sponge transplantation accelerate wound healing, enhance local capillary regeneration. • Own superior pro-angiogenic effects and enhance reepithelialisation.	(69)
**Composite scaffold**	Collagen	SDF-1α/VEGF	• This scaffold has a large surface area, avoids the side effects of high concentration caused by rapid diffusion and the mechanical properties of the collagen scaffolds are uniform.	• Scaffold promotes neovascularization by recruiting more vascular endothelial cells and reduces the macrophages accumulation at the injury site. • Co-modified functional collagen accelerates epidermal cell proliferation by promoting angiogenesis which could provide enough oxygen and other nutrients to wound bed and facilitates collagen deposition, maturation and integration.	(70)
	Chitosan/collagen	HBD-2 loaded poly nanoparticle	• High mechanical strength. • Better porosity, water absorption, collagenase degradation, and drug release.	• Scaffold accelerates reepithelialization, fibroblast, and keratinocyte migration. • HBD-2 reduces the protein expression of TNF-α, MPO enzymatic activity, MMP-9, NAG NO concentration and IL-10.	(71)
**Electrospun scaffold**	Collagen type I	Recombinant human dermal tropoelastin	• WHD results in remodeled skin that more closely resembles the non-wounded native skin in mechanics and architectural structure.	• WHD-treated wounds advances through the wound healing cascade at an accelerated rate resulting in wound resolution or skin regeneration. • It decreases tissue inflammation, accelerates wound closure, reconstruction of hair follicles and the reformation of a well-organized epidermis.	(72)
**Porous scaffold**	Fibrin	Allogeneic fibroblasts	• Biocompatibility, biodegradability, non-toxicity and non-immunogenicity.	• The added fibroblasts can accelerate the myofibroblast differentiation, collagen gene expression, and subsequent apoptosis and stimulate/inhibit the growth factors and promote wound bed maturation.	(73)
**Nanohybrid scaffold**	Collagen/alginate	Curcumin/chitosan nanoparticles	• The nanohybrid scaffold possesses porous morphology with good biodegradability and biocompatibility properties.	• CUR reduces the inflammation thereby promotes collagen deposition. • The nanohybrid scaffold supports cell adhesion, proliferation and decreases degradation of collagen at the wound site by CUR.	(74)
**Electrospun nanofibrous membrane**	Collagen/poly-D-L-lactide–glycolide	Glucophage	• High porosities, good hydrophilicity and water-containing capacity.	• The glucophage-loaded collagen/PLGA nanofibers scaffold enhances collagen content and the delivery of glucophage by PLGA nanofibers effectively decreasing MMP-9.	(75)
**Acellular dermal matrix**	Collagen binding domain	Histatin-1	• ADM shows a natural fluffy 3D fiber network and porous structure, and the collagen filaments are clearer and better organized and is a good slow-release carrier for C-Hst1.	• C-Hst1/ADM promotes the migration of HMECs, enhances the tube-forming activity of HMECs, and facilitates angiogenesis.	(76)
	Collagen fibrils	PA/Fe^3+^/HUVEC-Exos	• ADM has antioxidant and antibacterial properties. • Hydrogels exhibit rapid gelation, biocompatibility, and antioxidant properties	• The hydrogel improves the function of diabetic cells, inhibits bacterial growth, promotes collagen deposition, angiogenesis and maturation of diabetic wounds, and reduces oxidative stress and inflammation.	(77)

ADM, acellular dermal matrix; C-Hst1, collagen binding domain-histatin-1; CUR, curcumin; Exo, exosomes; HBD-2, human beta defensin-2; HUVEC, human umbilical vein endothelial cells; IL-1β, interleukin-1β; HMEC, human mammary epithelial cells; MMP-9, matrix metalloproteinase-9; MPO, myeloperoxidase; NAG, N-acetylglucosaminidase; PA, protocatechualdehyde; PDGF-BB, platelet-derived growth factor-BB; PLGA, polylactic-co-glycolic acid; sHA, high-sulfated hyaluronan; SDF-1α, stromal cell-derived factor-1α; TGF-β1, transforming growth factor-β1; TIMP1, tissue inhibitor of metalloproteinases-1; TNF-α, tumor necrosis factor-α; VEGF, vascular endothelial growth factor; WHD, wound healing device; SKP, skin-derived precursor.

### 4.1 Hydrogel

Hydrogels possess antibacterial, adhesive, hemostatic, anti-inflammatory, anti-oxidative, substance delivery, self-healing, stimulus-responsive, and conductive properties, making them highly advantageous for various wound dressings (78). Several investigations have shown that hydrogels can form a physical barrier and remove excessive exudate. They can create a moist environment to facilitate wound healing. In addition, hydrogels can be used as a sprayable or injectable wound dressing, attracting significant interest in diabetic wound dressing studies due to its ability to cover irregularly shaped wounds (79).

#### 4.1.1 Collagen-based hydrogels

One study revealed that the bonding of collagen hydrogels with fibroblasts can enhance skin thickness, density, and flexibility in wound areas by promoting collagen synthesis. This dressing also promotes angiogenesis, activates macrophages, and stimulates epithelial development (61). Similarly, a hydrogel containing immunomodulatory hyaluronan and collagen enhanced tissue repair by reducing inflammation, promoting pro-regenerative macrophage activity, increasing vascularization, and accelerating new tissue development and wound closure (62). Shagdarova et al. (63) developed a panel of hydrogels using chitosan, collagen, and silver nanoparticles for diabetic wound treatment. The study revealed that gels composed of 700 kDa chitosan and collagen had superior swelled properties compared to gels made only from collagen and 100 kDa chitosan. Applying the gels led to increased expression of VEGF, TGF-β1 and IL-1β genes and faster wound healing compared to untreated wounds. All gels enhanced collagen deposition, hair follicle restoration, and sebaceous glands development. The findings indicated that the hydrogels developed have promising potential for use in diabetic wound healing.

However, despite the high potential of metal nanoparticles in the treatment of drug-resistant bacteria, the high toxicity of these materials limits their application in wound healing.

In conclusion, these excellent properties of collagen can be utilized to prepare collagen-based hydrogels, which improves wound healing in diabetic patients, and at the same time has very low toxicity and side effects. But the three-stranded helical structure of collagen is not easily modified, and disruption of the helical structure leads to the loss of its original properties. Therefore, it would be better to strengthen the cross-linking network structure of collagen hydrogels and exploring the link between the structure and properties of collagen hydrogels (80).

#### 4.1.2 Other protein hydrogels

In addition to collagen, other fibrous proteins can be used as hydrogels, including fibrin, laminin, and the composite. Certelli et al. (64) studied the angiogenic and arteriogenic capabilities of engineered forms of VEGF and PDGF-BB proteins using a fibrin-based platform. They discovered that delivering both proteins simultaneously effectively promoted angiogenesis and arteriogenesis on the skin of mice with diabetes. But the positive effect is transient and most of the newly formed vessels degenerate after 4 weeks. Some patients with diabetic foot have prolonged treatment time due to recurrent wound infections, ranging from a few months to several years, implying that repeated treatments may be necessary to maintain the therapeutic effect, and therefore the fibrin hydrogel in this article needs to be further applied to the clinic to observe the therapeutic effect. In another literature, a gelatin-sericin hydrogel covered with laminin showed enhanced cell adhesion and then was used to load scaffolds with adipose-derived stem cells (ADSCs). ADSCs further showed a beneficial impact on wound healing by reducing inflammation and promoting collagen deposition. The impact of laminin coating was also noticeable in increased vascularization (66). In addition, Nilforoushzadeh et al. (65) were the first to report that the safety and wound healing benefits of full-thickness skin grafts with stromal vascular fraction cells enclosed in fibrin-collagen hydrogel, as opposed to commercially accessible dermal-epidermal skin grafts. Research has demonstrated that combining fibrin with collagen can promote the alignment of collagen fibers and regulate the release of angiogenic factors.

Overall, utilizing antimicrobial components from natural products has great potential for development due to its biocompatibility and lack of cytotoxicity. In addition, hydrogels are often subjected to external forces during use, resulting in hydrogel cracking and bacterial invasion (81). These fibrous proteins contain mucoadhesive functional groups, which enable hydrogels to interact and bind with surrounding tissues, adhering to the wound and preventing both dressing dislodgement and bacterial invasion, as well as rapidly blocking early bleeding from the wound.

### 4.2 Foam

Foams (also known as sponges) can absorb significant volumes of wound exudate and provide a moist environment at the wound site due to their biodegradability, porosity, and swelling characteristics (82). Collagen sponge consists of collagen which offers mechanical support and biological activity after implantation. It has been demonstrated to enhance mesenchymal stem cell differentiation and epithelial cell proliferation (69). Therefore, cutaneous wound dressing was developed with collagen sponge due to its above characteristics.

The study conducted by Varga et al. (67) aimed to evaluate the effects of the application of a gentamicin-collagen sponge on the surgical result of amputations in patients with diabetes. Gentamicin is initially released by the sponge by passive diffusion and subsequently actively by degradation of the sponge collagen. The sponge application reduced outpatient visits, decreased the amount of antibiotics used, and decreased the risk of complications from the systemic administration of antibiotics.

Ke et al. (69) employed collagen sponge for skin-derived precursors (SKPs) better delivery, which have been shown to differentiate into vascular and nerve cells. In comparison to SKPs that were cultured without collagen sponge, the outcomes demonstrated that SKPs that were co-cultured with collagen sponge had higher cell survival, better wound gap closure and collagen deposition, and more rapid wound healing.

Compared with collagen based-hydrogel, collagen-based foams possess anti-adhesion ability, are degraded by collagenase degradation into peptides and amino acids in 1-2 weeks, and can be completely absorbed in 4-6 weeks. But because they are made of opaque material, it is not easy to observe the wound condition, and it is not easy to be fixed when using non-adhesive products in some wound locations.

### 4.3 Scaffold

Scaffolds-3D structures-play a pivotal role in wound-healing treatment. In addition to providing sustenance for the development of new tissue, they are special in that they aid in tissue regeneration and repair by offering a proper platform that makes it possible for multiple factors necessary for cell survival, proliferation, and differentiation to be supplied (83). Scaffolds should be both porous and biocompatible since cells need to adhere and move through their networks (84). They can consist of synthetic or absorbable, naturally occurring, biodegradable or non-biodegradable polymeric materials (66). Below we have listed the common wound dressings that bind to fibrous proteins (**Table 2**).

**Table 2:** Comparison of different types of scaffold.

Types of scaffolds	Advantages	Disadvantages
COL-chitosan composite scaffold	• COL makes up for the defects of simple chitosan scaffolds with slow degradation, inability to adsorb and poor intracellular attachment ability, promotes cell value-added differentiation and has good mechanical properties.	• COL may be more costly, limiting their widespread use. • Chitosan is hydrophobic while collagen is hydrophilic, and a balance of hydrophilicity and hydrophobicity is required when compounding the two to ensure cytocompatibility and promote cell growth.
Tropoelastin-collagen electrospun scaffold	• Excellent elasticity and flexibility for cell adhesion and proliferation. • Adjusting electrospinning process parameters (e.g., voltage, flow rate, receiving distance, etc.) can tailor fiber diameter, orientation and porosity.	• Extraction and purification of raw elastin and collagen are costly, affecting the economic viability of the scaffolds.
Fibrin membrane-porous scaffold	• Has an open and interconnected porous structure with a certain mechanical strength. • Provides a physical surface for cells to bind and generate ECM. • The interconnected pores provide a nutrient supply to the center of the device, reducing the incidence of central necrosis. • Does not stimulate an immune system response or an inflammatory response to wounds.	• The preparation process can be complex and requires fine control of the pore structure. • The rate of degradation of the scaffold may not be easy to control, affecting the wound healing process.
Alginate-collagen nanohybrid scaffold	• Alginate improves mechanical properties and collagen can provide cell adhesion sites. • Has high porosity, which facilitates cell migration and nutrient transport.	• The higher cost of collagen extraction compared to synthetic materials restricts scaffold widespread use and makes its production on a clinical scale somewhat. limited.

#### 4.3.1 Composite scaffold

Stromal cell-derived factor-1α (SDF‐1α) is a C-X-C motif chemokine ligand 12 that performs various biological functions, including stem cell migration, inflammatory cell infiltration, and angiogenesis, all of which are essential for wound healing (85). At the site of injury, SDF-1α gradients can be established by the local inflammatory microenvironment and have a role in directing the migration of circulating bone marrow stem cells (BMSCs), suggesting that even direct application of SDF-1α to the site of injury has a therapeutic effect (86). VEGF is a special factor in fibroblast cell migration that stimulates angiogenesis, collagen deposition, and epithelialization (87). One typical approach to accelerate the healing process of diabetic wounds is to deliver chemokines and growth factors to the wound site using natural and synthetic dermal substitutes or biomaterials. Thus, Long et al. (70) prepared a collagen membrane as a drug delivery scaffold. They fused a collagen-binding domain (CBD) with SDF-1α and VEGF respectively, and the two recombinant proteins were successfully shown to release from the collagen scaffold. After implantation of CBD-VEGF and CBD-SDF-1α co-modified scaffold in a diabetic rat skin wound model, it was observed to have a synergistic impact on promoting angiogenesis and temporarily lowers inflammation. Moreover, long-term results also showed that the co-modified scaffold can help blood vessel regeneration, increase wound healing, and support cell proliferation, re-epithelialization, and ECM buildup.

Human beta defensin-2 (HBD-2) are crucial for better wound healing with the anti-inflammatory, cell-proliferating, migratory, and angiogenic qualities. Despite the fact that diabetic wounds produce HBD-2, reports indicate that the inadequate expression of them contributes to impaired wound healing (88). Thus, a novel topical formulation of HBD-2 is required to manage diabetic wound. Polylactic-co-glycolic acid (PLGA) is a widely recognized material in nanotechnology because of its biodegradability and biocompatibility properties. It is frequently employed in drug delivery applications, wherein the combination of drugs and inorganic nanomaterials can enhance the effects of the medications and provide the particles additional capabilities (89). Chitosan (CS) has emerged as a promising material for wound healing applications due to its distinct biological characteristics, such as its biocompatibility, biodegradability, and low toxicity. Furthermore, it possesses mucoadhesive, hemostatic, and antimicrobial qualities and may also fasten the healing of wounds (84). Taken together, Sanapalli et al. (71) developed HBD-2 loaded PLGA nanoparticle impregnated in collagen-chitosan (COL-CS) composite scaffolds. The HBD-2 COL-CS scaffold was shown to be biocompatible and to promote angiogenesis and cell migration *in vitro* experiments. According to *in vivo* studies, the accelerated healing in the HBD-2 COL-CS treatment group was the result of the combined benefits of the above mentioned things: PLGA is associated with collagen synthesis and deposition and positive angiogenic effect; HBD-2 is anti-inflammatory, antibacterial, and promote cell proliferation and migration; COL acts as established wound healer and stabilizer and CS can control drug release.

#### 4.3.2 Electrospun scaffold

Electrospinning is a well-established technique used to produce nanoscale fibers. Researchers are interested in this technology because it is seen as the most practical method for creating advanced scaffolds that can be used in wound healing (90). Polymeric electrospun scaffolds offer a 3D support for cell adhesion, migration, proliferation, and differentiation. Various natural and synthetic polymers have been used for tissue engineered scaffolds (91). In the study of Kellar (72), an electrospun biomimetic scaffold, wound healing device (WHD) that contained tropoelastin (TE) and collagen were prepared. This device was designed to imitate the biochemical and mechanical properties of healthy human skin and demonstrated the characteristics of normal wound healing, including reduced tissue inflammation, faster wound closure, regeneration of hair follicles, and the formation of a well-structured epidermis, leading to remodeled skin that closely resembles uninjured skin in terms of mechanics and architecture. However, it remains uncertain whether electrospun biomimetic scaffold affects cell behavior and tissue regeneration processes. Further studies are needed to investigate drug selection, loading and delivery methods, dosage and preclinical animal model studies. Although only a few *in vivo* studies to date have demonstrated the clinical potential of nanofiber scaffolds, most studies have been exploratory and relied on *in vitro* experiments. Therefore, more studies are needed for application to clinical treatment.

#### 4.3.3 Porous scaffold

The porous scaffolds can facilitate cell seeding and nutrient exchange, then such cell-seeded porous grafts are used for implantation into hosts. They are available in a variety of forms, including sponge, mesh, and biodegradable fibers at the nano- and microscale. Furthermore, these scaffolds possess interconnected pore networks with increased porosities to mimic ECM formation, promoting effective cell contact with their surroundings (83). A fibrin membrane is a biocompatible and biodegradable porous scaffold that has advantages like availability, flexibility, high seeding efficiency, adhesion capability, and no risk of foreign body reaction or infection. Consequently, a cellular fibrin membrane was employed to assess the process of diabetic wound healing in rats in the study of Kouhbananinejad et al. (73). The fibrin membrane was created by culturing isolated fibroblasts over fresh frozen plasma. The use of an elastic cellular fibrin membrane containing allogeneic fibroblasts can modulate the growth factors and promote wound bed development. More importantly, the fibrin membrane, whether cellular or non-cellular, does not stimulate immune system responses or inflammation at the wound site. It can serve as an effective skin substitute for treating diabetic wounds.

#### 4.3.4 Nanohybrid scaffold

Nanohybrid materials are composite materials formed by mixing nanoparticles with other materials. They have been applied in wound healing in various literatures, for example, Liu et al. (92) proved that nanohybrid dual-network chitosan-based hydrogels possess qualities such as injectability, stability, self-healing, and adhesion. They can help decrease bacterial infections, enhance cell migration and angiogenesis, and lower the release of inflammatory factors. These hydrogels demonstrate exceptional healing capabilities in infected full-thickness wounds; NIR-II responsive nanohybrids incorporating thermosensitive hydrogel revealed excellent bacteria eradication and wound repair benefits (93); The polyvinyl alcohol-alginate nanohybrid demonstrated enhanced mechanical capabilities, adjustable degradation rate, excellent biocompatibility, and facilitated hemostasis, making it beneficial for wound dressing (94). Therefore, based on the above facts, nanohybrid would be a promising tool in promoting diabetic wound healing.

Collagen’s primary drawback as a scaffold is its biological instability. To improve collagen’s mechanical strength and prevent its degradation, chemical cross-linking or combining it with synthetic polymers or natural polysaccharides are considered effective methods for fabricating collagen-based scaffolds with enhanced properties. Karri et al. (74) prepared a nanohybrid scaffold by blending alginate with collagen and then cross-linking it to enhance its physical stability and create a moist wound environment. They then added curcumin-chitosan nanoparticles (CUR-CSNPs) to collagen-alginate scaffolds. The use of this nanohybrid scaffold on the skin helped stimulate the healing of wounds by reducing inflammation at the wound site in diabetic rats. However, they only studied the phenotypes and did not mention the pathways or mechanisms in the wound healing process. Therefore, deeper studies could be done to minimize or avoid side effects during the healing process.

### 4.4 Electrospun nanofibrous membrane

Electrospinning nanofiber membrane is a type of nanostructured material created by electro-spinning technology. It consists of nanofibers with a diameter below 1000 nm that interact to form a web structure. They possess a large specific surface area, high porosity, minuscule pore size, and adjustable composition, structure, and size (95). Electrospun polymeric nanofibers are considered beneficial for enhancing diabetic wound healing due to their similarity to ECM of normal skin, capacity to promote cellular growth and proliferation, bactericidal properties, and capability to transport bioactive molecules to the wound location (96). Lee et al. (75) created nanofibrous collagen/PLGA scaffold membranes loaded with glucophage to deliver the drug gradually for diabetic wound treatment. The collagen/PLGA membranes containing glucophage notably improves the healing process. This study findings indicate that the above membranes effectively elevated collagen concentration and significantly promoted the healing of diabetic wounds in the early stages.

The preparation of nanofiber collagen/PLGA scaffold membranes using the emerging electrostatic spinning technology not only combines the unique skin-healing-promoting properties of collagen with the structural advantages of nanofiber membranes, but also serves as a delivery vehicle for different drugs, which has significant potential for promoting wound healing and skin repair, and is expected to be developed as an ideal wound dressing. However, the parameters affecting the morphology and structure of nanofibers are complex and varied. The stability of the electrostatic spinning process is difficult to be controlled (97). So far, the clinical application of electrospun nanofibrous membrane as wound dressings has been seldom reported, and there are no uniform indexes and imperfections regarding the performance parameters and evaluation methods.

### 4.5 Acellular dermal matrix

The acellular dermal matrix (ADM) is obtained by decellularizing the cells and preserving collagen fiber scaffolding and basic tissue structure based on the composition and structure of the ECM. It has been prevalent in the realm of soft tissue replacement, particularly in situations involving wound healing. Literature has found that the use of ADM showed superiority over the standard of care alone, without causing any complications, and it has the ability to enhance the rate of healing for uninfected, non-ischemic, full-thickness diabetic foot ulcers (98) and prevent degradation of the ECM. Healing rates of up to 80% with the application of ADM materials. Based on the above facts, ADM is ideal for skin tissue scaffold that facilitates cellular migration, proliferation, and the formation of endogenous matrix (99). Aiming at the clinical problem of angiogenesis disorder in diabetic wounds, CBD-Histatin-1 (C-Hst1) was incorporated into a new acellular dermal drug sustained-release scaffold. Wound healing studies showed that the sustained release of Histatin-1 by C-Hst1/ADM can promote the adhesion, migration and angiogenesis of vascular endothelial cells. C-Hst1/ADM has a good effect on promoting the angiogenesis of diabetic wounds, thus promoting the reduction of scar width and the deposition of extracellular collagen, and promoting the rapid wound healing. This study suggested that C-Hst1/ADM sustained-release stents could provide a new strategy for clinical diabetic wound treatment (76). Xiang et al. (77) combined ADM with protocatechualdehyde (PA) and Fe^3+^ complex, exosomes derived from human umbilical vein endothelial cells (HUVEC-Exos) and GelMA to make hydrogel. This ADM owns antioxidant and antimicrobial properties. The experiment proved that the hydrogel can effectively improve the function of diabetic cells, inhibit bacterial growth, promote collagen deposition, angiogenesis and maturation of diabetic wounds, and reduce oxidative stress and inflammation. It provides a new possibility for the clinical method of ADM in diabetic wounds.

Compared with the above dressings, ADM retains the structure and bioactivity of the natural extracellular matrix. It promotes the formation of new functional dermal tissue by providing a bioactive environment. While it offers a healing environment that is more in line with the body’s natural processes, it may come with a higher price (100).

## 5 Clinical trials within last 5 years

Clinical trials of wound dressing applications over the past five years were searched on clinicalTrials.gov using the keywords “wound dressings” and “diabetic foot ulcers.” (101) The initial search yielded 88 clinical trials, of which 24 completed trials were screened. The number of ongoing clinical trials of wound dressings demonstrates the growing interest of researchers in the field of diabetic wound care. Of the 24 completed trials, 20 used modern wound dressings such as hydrogels, stents, and laser treatments, of which only 3 described fibrous proteins-based wound dressings. Therefore, the combination of fibrous proteins with modern wound dressings could be considered for clinical use.

The use of modern wound dressings as described above for clinical use is thought to also require the following considerations. First is about the above studies. It was found that most of the articles had a small number of animal samples thus affecting the ability to extrapolate the results to human diabetic wound healing and most of the animals are rats and mice due to differences in animal cost, size and availability. However, the skin morphology and wound healing processes in these rodents are different from those in humans. In contrast, pig skin is most similar to human skin. Pigs are not widely used in wound healing studies due to the high cost and cumbersome nature of large animal experiments (102). As they are all short-term trials, they do not provide long-term follow-up data, which can affect the assessment of the sustained effects of wound healing and thus the clinical application.

Second is about these proteins. Although fibrous proteins are natural proteins and are compatible with human tissue, it is important to consider whether these proteins cause immune and tissue reactions, especially in the case of xenografts.

The last one is selection. When treating diabetic wounds, the selection of the appropriate dressing type should be based on the patient’s type of wound, the stage, and individual condition. Suitable types of dressings should be selected according to the above. For example, collagen foams are suitable for wounds with moderate amount of exudate, which can absorb wound exudate and protect the wound surface; fibrin composite scaffolds are used to deliver growth factors, stem cells and other materials, which can promote the wound healing more quickly, and so on. Overcoming the above difficulties will be of great help to the application of wound dressings in the clinic.

## 6 Perspectives and conclusions

In summary, this paper summarizes the impact of fibrous proteins on diabetic wound healing. These proteins are widely used in wound repair because of their good biocompatibility and degradation. However, currently collagen has been studied in depth regarding the mechanism in DFU, while several other proteins are only known to have their specific roles. Diabetic wound healing is a complex process involving the interactions of multiple cells, cytokines, GFs, and the ECM. So more in-depth studies are needed to investigate the specific mechanisms of their roles in DFU, and to delve deeper into how these proteins affect the synthesis, modification, degradation, and function of ECM in the diabetic environment and how they influence other cytokines or growth factors to interact with ECM proteins to promote DFU.

Due to the patient’s age, health, and the size and shape of the wound, personalized dressings may be a future treatment target. Therefore, further trials are needed on more fibrous protein-based wound dressings to explore their clinical applications in the wound. Future research on fibrous protein-based biomaterials can consider using smart hydrogel dressings that can monitor wound status (e.g. glucose levels) in real time. The hydrogel dressing developed by professor Zhang (103) is able to convert pH and glucose signals from diabetic wounds into optical signals and quantify the data via cell phone. Designing nanoreactor hydrogels based on metal-organic framework compounds (MOFs) can also be considered. These dressings can respond to high glucose localized in wounds by lowering pH while generating therapeutic molecules such as NO for antibacterial and anti-inflammatory purposes.

In addition, owing to the challenges of extracting these proteins and their expensive costs, it is probable that fibrous protein derivatives will be developed into medical products for commercial use in promoting wound healing due to their benefits of improved bioactivity, reduced cost, and increased controllability, offering novel approaches for clinical treatment.

There are some limitations in this article. Most of the literature cited are animal experimental articles, with little clinical relevance, making it challenging to translate from basic research to clinical application; The biological mechanisms of diabetic wound healing are complex, and the review may not have fully revealed all the relevant mechanisms; Due to the large number of types of wound dressing involved, it failed to thoroughly introduce the material manufacture and characteristics of wound dressings.

## Author contributions

MZ: Funding acquisition, Visualization, Writing – review & editing. LY: Writing – original draft, Writing – review & editing. YW: Writing – review & editing. JF: Writing – review & editing. YN: Writing – review & editing. TZ: Writing – review & editing. YC: Writing – review & editing. CZ: Writing – review & editing.
